# An automated deep learning framework for brain tumor classification using MRI imagery

**DOI:** 10.1038/s41598-025-02209-2

**Published:** 2025-05-21

**Authors:** Muhammad Aamir, Ziaur Rahman, Uzair Aslam Bhatti, Waheed Ahmed Abro, Jameel Ahmed Bhutto, Zhonglin He

**Affiliations:** 1 School of Computer Science and Artificial Intelligence, Huanggang Normal University, Huanggang, 438000 Hubei China; 2 School of Information and Communication Engineering, Hainan University, Haikou, 68000 China; 3 Artois University, Arras, France

**Keywords:** Healthcare, Convolutional neural network (CNN), Brain tumor classification (BTC), Image enhancement, Morphological process, MRI Segmentation, Multiscale feature extraction, Attention mechanism, Ensemble classifier, Image processing, Brain imaging

## Abstract

The precise and timely diagnosis of brain tumors is essential for accelerating patient recovery and preserving lives. Brain tumors exhibit a variety of sizes, shapes, and visual characteristics, requiring individualized treatment strategies for each patient. Radiologists require considerable proficiency to manually detect brain malignancies. However, tumor recognition remains inefficient, imprecise, and labor-intensive in manual procedures, underscoring the need for automated methods. This study introduces an effective approach for identifying brain lesions in magnetic resonance imaging (MRI) images, minimizing dependence on manual intervention. The proposed method improves image clarity by combining guided filtering techniques with anisotropic Gaussian side windows (AGSW). A morphological analysis is conducted prior to segmentation to exclude non-tumor regions from the enhanced MRI images. Deep neural networks segment the images, extracting high-quality regions of interest (ROIs) and multiscale features. Identifying salient elements is essential and is accomplished through an attention module that isolates distinctive features while eliminating irrelevant information. An ensemble model is employed to classify brain tumors into different categories. The proposed technique achieves an overall accuracy of 99.94% and 99.67% on the publicly available brain tumor datasets BraTS2020 and Figshare, respectively. Furthermore, it surpasses existing technologies in terms of automation and robustness, thereby enhancing the entire diagnostic process.

## Introduction

Today, specialists are inundated with various medical data, including electronic health records, patient medical histories, and test results. However, the ability to evaluate, accumulate and manage such vast amounts of data is restricted, raising concerns about the potential for fatigue to impede the ability of healthcare professionals to assist their patients and affect their health. This presents a substantial obstacle for the healthcare sector, as providing accurate and timely diagnoses is imperative to achieve favorable patient outcomes^1^. The processing of medical images is essential for planning treatment and other purposes, as they comprise 90% of healthcare data. As a result, there is an increasing demand for medical image analysis, which presents a significant opportunity to develop innovative IT-based healthcare solutions^2^. These technologies can improve the diagnostic experience of a patient by reducing the duration of the diagnostic process, improving the precision of the diagnostic, and helping hospitals and medical professionals optimize their operations^3^.

Image processing, a fundamental aspect of computer vision technology, has attracted considerable interest in the healthcare sector due to its diverse applications and continuous advancements^4^. In numerous healthcare specialties, including orthopedics^5^, neurology^6^, dentistry^7^, cardiology^8^, and oncology^9^, image processing techniques such as enhancement^10^, segmentation^11^, detection^12^, and classification^13^ are widely recognized and commonly applied for the diagnosis of disease. The capability of these image processing technologies to reliably assess and extract features from images obtained through various imaging methods significantly enhances the effectiveness and accuracy of disease diagnosis. These technologies show considerable effectiveness, allowing physicians to identify and diagnose irregularities within images^14^.The healthcare sector has undergone significant changes due to the progress in medical imaging technology, offering a non-invasive approach to examine and gather accurate information about different parts of the body, thus facilitating the diagnosis of a wide range of diseases. Computed tomography (CT), positron emission tomography (PET), ultrasound imaging (UI), magnetic resonance spectroscopy (MRS), single-photon emission computed tomography (SPECT) and MRI are the most widely used techniques^15^. Each of these methods has its own distinct advantages and disadvantages, which make them suitable for specific medical conditions. MRI is the most widely used and valuable technology, as it can provide a wealth of information on the anatomy of the human body ^16^. Brain tumors are among the most complicated and dangerous conditions that affect the human brain. A tumor is an abnormal mass resulting from excessive cell division and is one of the leading causes of death worldwide^17^. Brain tumors are commonly classified based on their location and behavior, i.e., benign or malignant, low grade or high grade, cancerous or non-cancerous, and tumors that are found in specific areas such as the pituitary gland. Brain tumors are heterogeneous in size, location, form, and type, complicating diagnosis and therapy^18^. MRI, the most reliable and common approach for the detection of brain malignancies, detects any kind of brain tumor and provides high-contrast images. It is the most used approach for classifying brain tumors and provides high-resolution imaging with detailed information^19^. Correct classification of brain tumors is crucial for favorable patient outcomes, as misdiagnoses will ultimately lead to ineffective therapy and a drastic decrease in patient quality of life. A timely and accurate diagnosis is essential for effective treatment planning because a delayed diagnosis can worsen the disease and reduce survival rates^20^.

However, BTC is a challenging task as the imaging characteristics of brain tumors are unpredictable and there are many different types of tumor. Although many frameworks have been developed to diagnose brain lesions in MRI images recently, most do not reach satisfactory classification performance^21^. Traditional approaches typically rely on hand-designed features that are labor intensive, susceptible to subjective bias, and cannot generalize to various datasets. Although deep learning models are much more advanced, they still struggle with limited training data, overfitting, and managing the large variation in tumor shape, size, and imaging characteristics. Issues such as challenging tumor locations, substandard image segmentation, and low-quality image features can significantly degrade the accuracy and reliability of classification systems. Therefore, much research should be done to construct more efficient classifiers to help patients with accurate and precise diagnoses. Building a classification algorithm that can handle these issues simultaneously while delivering state-of-the-art performance is a challenging endeavor. This work will ultimately contribute to the advancement of ongoing research, helping researchers, academics, and practitioners continue to gather evidence to clarify the link between BTC and patient survivability, which is necessary to suggest better treatment plans and improve patient health.

The system under consideration can significantly improve computer-aided diagnosis (CAD) and alleviate the burden on healthcare professionals, thus resolving resource imbalances and physician-patient disputes. Because BTC in medical imaging involves several technical considerations that could significantly affect medical diagnosis and treatment, these difficulties must be addressed. However, interdisciplinary research on medical image classification is still in its infancy.  Although the accuracy of medical diagnosis depends on classification accuracy, the technological challenges surrounding medical image categorization are still being investigated. These challenges directly affect the way computer science is applied in the medical field. More study and development are needed to overcome these obstacles and guarantee that computer science can be applied to medical picture classification efficiently, improving patient outcomes. The primary contribution of this work is detailed as follows:

- The overall accuracy of the classification is enhanced by the proposed automated technique to supplant the traditional invasive classification of brain tumors.
- A preprocessing strategy is used to enhance the quality of brain tumor images by integrating guided filtering methods with AGSW.
- To obtain a high level of classification accuracy on a limited dataset, data augmentation techniques are used, and the impact of overfitting on classification performance is also investigated.
- Deep learning-based segmentation is employed to extract high-quality ROIs from MRI images, enhancing the categorization capability.
- To enhance the contextual connection between the discriminative representation and the extracted multiscale features, an attention method is employed.
- Ensemble learning algorithms are used to categorize brain tumors and the classification accuracy is improved by integrating multiple classifiers.
- The proposed solution is being assessed compared to the established advanced BTC algorithms. The classification accuracy of the proposed technique surpasses that of existing methods.

The remaining parts of the paper are organized as follows: “Related work” section reviews the research on BTC. The proposed paradigm is discussed in more detail in “Proposed methodology” section. “Evaluation and results” section presents and analyzes the results of the experiments. This study concludes with suggestions for further research in “Model interpretability, trustworthiness, and generalization across datasets” section.

## Related work

The classification of brain tumors is a significant and well-researched topic in medical image analysis due to the importance of timely and accurate diagnosis for patient safety and treatment planning. The preferred method for classifying brain tumors is automated techniques, which are efficient, accurate and require minimal human involvement. The use of machine learning in the development of automated disease diagnosis systems has expanded, particularly in the context of newly induced brain tumor categorization techniques. Traditional machine learning- and deep learning-based methods are the two varieties of machine learning-based methods that are used to classify brain tumors in MRI images. Preprocessing, localization, feature extraction, and classification are required for both methods. Classical machine learning-based algorithms require the extraction of hand-made features, which affect classification performance, to achieve high classification accuracy. In contrast, deep learning-based methodologies outperform conventional machine learning-based methodologies. This is because deep learning architectures, such as CNN and its variants, leverage their ability to generalize and learn independently to offer automatic and reliable quantitative analyses of image attributes. A greater amount of training data is required, as the capacity to generalize may be impaired by the limited number of datasets and low-resolution images. To achieve high classification accuracy, this study closely examines each stage of the brain cancer classification process and provides a concise overview of recent research endeavors and work conducted to classify brain tumors using MRI images and the corresponding issues. Significant advances in the classification of brain tumors are presented in Table 1.

### Traditional machine learning-based BTC methods

The standard machine-learning classification of brain tumors using MRI images involves several fundamental procedures, including preprocessing, localization or segmentation, feature extraction, and classification. It is imperative to analyze the unique contributions of each stage in the BTC process to achieve effective classification performance. Feature extraction is one of the primary components contributing to these models’ enhanced classification accuracy. Local feature extraction (low-level features) and global feature extraction (high-level features) are the primary categories of traditional feature extraction methods. Most local features (low-level) are composed of approaches based on wavelet transform, symmetry, texture, intensity, Gabor feature, and shape. These techniques employ statistical features for feature extraction, such as the mean, standard deviation, skewness, and grey level co-occurrence matrix (GLCM). In contrast, global features (high-level) such as scale-invariant feature transformation (SIFT), fisher vector (FV), and bag of words (BoW) capture broader, more abstract representations of the image. The accumulated features are subsequently fed into machine learning-based classifiers, including Naive Bayes, support vector machine (SVM), random forest (RF), and artificial neural network (ANN), to determine the type of tumor. In this regard, the subsequent section examines a variety of methodologies that have been developed and are currently under investigation: Othman et al.^22^ proposed a model for classifying brain MRI images that utilizes wavelet transform and SVM features. The model extracted 17,689 feature vectors from a single MRI image and categorized 39 out of 60 images with 65% accuracy. Sindhmol et al.^23^ published a BTC model that employs SVM for classification, independent component analysis (ICA) for enhanced feature extraction, and the spectral distance technique for aggregating the MRI image. The model achieved a 98% accuracy rate in identifying 40 normal and 20 aberrant brain MRI images. Similarly, Abd-Ellah et al.^24^ employed the discrete wavelet transform to extract features and subsequently employed SVM to identify brain tumor images from MRIs. A small sample of 80 photographs was used to test the efficacy of 32 MRI pictures, and the model achieved 100% accuracy. Kalbkhani et al.^25^ developed a brain MRI image classification model using 2D DWT coefficients and a generalized autoregressive conditional heteroscedasticity (GARCH) statistical approach. These were refined through linear discriminate analysis (LDA) and principal component analysis (PCA). For two distinct cases, the accuracy of the model was 97.62% and 98.21%, respectively, when using KNN and SVM classifiers. Saritha et al.^26^ employed the wavelet transform to extract features from MRI images, and probabilistic neural networks were employed for classification. After completing 50 MRI scans of training, the model was assessed on 23 scans and yielded 100% accurate results. Deepa et al.^27^ developed an artificial neural network for tumor classification using MRI images. The network was classified using the radial basis function (RBFN) and back-propagation network (BPN) after retrieving textural statistical features. The RBFN model achieved an accuracy of 85.71% after being trained on 30 images and tested on 12 images. Chandra et al.^28^ constructed a BTC model that was based on particle swarm optimization (PSO) and utilized GLCM features extracted from MRI scans. The image was divided into multiple clusters until they were merged into a single cluster. The model achieved an accuracy of 94.42% when trained on a dataset consisting of 110 aberrant brain MRI images and 62 normal brain MRI images. Xuan et al.^29^ developed a segmentation-based BTC model using symmetry-, texture-, and intensity-based features extracted from MRI images. The most effective features were selected to identify the class of MRI images, as evidenced by a 96.82% accuracy rate on 24 segments of MRI images from 10 patients. Cheng et al.^30^ developed a model for enhancing the classification performance of brain tumors by augmenting and partitioning tumor regions. The model’s various features, such as BOW, intensity histogram, and GLCM, were tested on a 3064 brain MRI images dataset. The Smythe model was employed to classify the results, achieving an accuracy of 91.28%. Ismael et al.^31^ introduced a neural network-based model for BTC that employs 2D DWT and 2D Gabor filter features. The classification performance was enhanced, and 91.9% accuracy was achieved by integrating these statistical characteristics. Tahir et al.^32^ developed a model for the classification of brain tumors by utilizing MRI imaging. Daubechies wavelets were employed to derive 2D DWT features, and SVM was employed for classification. The model ensured classification accuracy by demonstrating an accuracy of 86% on a dataset of 3064 brain MRI images.

### Deep learning-based BTC methods

It has been observed that conventional machine learning-based methods for BTC, which rely on manually generated features, tend to produce significantly poorer classification results. However, deep learning-based techniques overcome this limitation by autonomously extracting characteristics through self-learning, which enhances accuracy. Conversely, these models necessitate a more extensive dataset and substantial computational expenditures. The classification performance and accuracy are reduced when working with a limited dataset. Additionally, a high level of expertise is required for the practical application of deep learning model design and selection for a specific task. To overcome these challenges, a variety of strategies have been developed. For instance, Paul et al.^33^ introduced a deep learning-based model for classifying brain lesions that employ a CNN to enhance classification accuracy. The model’s accuracy on brain tumor imaging after five-fold cross-validation (5-fold CV) was 90.26% . This suggests that the efficacy of training can be improved and that physicians can more effectively treat patients by reducing the scale of the image. Similarly, Afshar et al.^34^ developed a capsule network (CapsNet) model for effectively categorizing brain tumors. This model enhances classification accuracy by utilizing spatial relations between the tumor and adjacent tissues, a limitation of previous CNN-based classification methods. Their model outperforms previous counterparts ^11,12^ ^17,18^ by achieving an accuracy of 86.56% and 72.13%, respectively, with and without segmentation. Furthermore, Afshar et al.^35^ proposed a modified capsule network (CapsNets) to classify brain lesions that circumvent the limitations of CNN. Their model is more robust than CNN because it does not require significant training data and can accommodate input modifications such as rotation and affine transformation. Outperforming its competitors, this model achieved a classification accuracy of 90.89%. Zhou et al.^36^ enhanced classification accuracy by employing a comprehensive strategy. Using a recurrent neural automated segmentation of regions technique, they utilized a dense convolutional neural network (DenseNet) to categorize the characteristics derived from axial slices of images. The high accuracy of 92.13% of their model serves as evidence of its efficacy. Pashaei et al.^37^ developed a CNN-based model for BTC in a similar manner. This method employs a CNN to extract features and a kernel extreme learning machine (KELM) network to classify images based on these characteristics. The experimental results of this joint-based mechanism of CNN and KELM are promising in terms of accuracy, with a score of 93.68%, when compared to other traditional machine learning classifiers such as radial basis function neural network (RBFNN), k-nearest neighbor (KNN), and SVM. In addition, Abiwinanda et al.^38^ have created a CNN model for classifying brain lesions. They generated seven distinct CNN iterations without segmentation. Their second variant exhibited the highest training and testing accuracies compared to their predecessors, with values of 98.51% and 84.19%, respectively. Ghassemi et al.^39^ introduced an additional multiclass BTC model based on deep neural networks. Using data augmentation techniques, their model can extract features and learn the structure of images by pretraining a neural network as a discriminator in a generative adversarial network (GAN). The augmentation strategies prevent the network from overtraining. The model is trained to function as a classifier to differentiate between the tumor types, and the wholly connected layers of the network have been replaced. The model’s accuracy was 95.6% and 93.01% for inserted and random divisions, respectively, when using 5-fold CV criteria. Furthermore, investigations have investigated using CNN in the graph domain for tumor classification. Guo et al.^40^ have introduced a graph CNN model for the prognosis of Alzheimer’s disease based on positron emission tomography (PET). Their model has demonstrated robustness on the alzheimer’s disease neuroimaging initiative (ADNI) dataset, unlike other cutting-edge models. It has achieved an accuracy of 93% for two-class classification problems and 77% for three-class classification problems. Furthermore, their computational paradigm is relatively cost-effective. Anaraki et al.^41^ examined the potential of genetic algorithms to enhance CNN’s capacity to classify brain lesions. They employed a genetic algorithm to optimize the design of CNN in their study and tested it on the Figshare dataset (https://figshare.com/articles/braintumordataset/1512427), achieving an accuracy of 94.2%. Nevertheless, the genetic algorithm could not select the optimal CNN architecture, resulting in sub-par accuracy. In an endeavor to enhance the efficacy of tumor classification, Ayadi et al.^42^ have proposed a deep CNN with multiple layers for BTC. Their model demonstrated exceptional performance when assessed on three datasets and necessitated significantly less pre-processing than previous methodologies. In an analogous vein, Deepak et al.^43^ have implemented transfer learning to enhance the precision of their three-class BTC. Their model outperformed other existing methods by obtaining a classification accuracy of 97.10% with a restricted number of training instances. Additionally, their model investigated the phenomenon of misclassification. Sejuti et al.^44^ created a CNN classifier to categorize different brain cancers. The classifier is trained using a dataset of 3064 photographs, which are divided into three distinct groups to represent different types of tumor images. The research work’s final efficiency was determined to be 97.1%. Kumar et al.^45^ created a sophisticated neural network model to tackle the problems of overfitting and vanishing gradients. This model utilizes global average pooling and ResNet50. When data augmentation was utilized, the study achieved an accuracy of 97.08%, compared to a slightly higher accuracy of 97.48% without data augmentation. Kakarla et al.^46^ proposed employing a CNN with eight average-pooling layers to categorize brain lesions into three groups. This model consists of a softmax layer and a dense layer, which are coupled to three convolution blocks. To improve the learning speed and achieve an accuracy of 97.42%, they used a sparse-categorical cross-entropy loss function and a Nesterov-accelerated adaptive moment estimation (Nadam) optimizer.

Despite these advancements^47^, existing models and research techniques still have several drawbacks. This work seeks to solve this issue by developing a highly accurate classifier for BTC using MRI images.

**Table 1:** A review of current approaches for BTC and their effectiveness.

Study	Method used	Dataset	Key contributions	Advantages	Limitations	Computational efficiency
Othman et al. (2011)	SVM with Wavelet Features	60 MRI images	Extracted 17,689 feature vectors per image	Good feature extraction	Low accuracy (65%), small dataset	Moderate
Sindhmol et al. (2013)	ICA + SVM	60 MRI images	98% accuracy with spectral distance technique	Effective with small datasets	Limited dataset, lacks deep learning automation	Low
Abd-Ellah et al. (2016)	Discrete Wavelet Transform + SVM	80 MRI images	100% accuracy on a small dataset	High accuracy on limited data	Not tested on large datasets	Moderate
Kalbkhani et al. (2013)	2D DWT + GARCH + LDA + PCA	3064 MRI images	Achieved 97.62% accuracy with SVM	Strong statistical feature extraction	Complex preprocessing, computationally expensive	High
Saritha et al. (2013)	Wavelet Transform + Probabilistic Neural Networks	50 MRI images	100% accuracy	Good for pattern recognition	Small dataset, lacks generalization	Moderate
Deepa et al. (2012)	ANN + RBFN	30 training, 12 testing	85.71% accuracy	Fast training	Low accuracy, limited dataset	Low
Chandra et al. (2009)	Particle Swarm Optimization (PSO)	110 abnormal, 62 normal MRI images	94.42% accuracy	Good optimization approach	Complex model, may not generalize well	High
Xuan et al. (2007)	Segmentation + Statistical Features	10 patient MRI images	96.82% accuracy on selected MRI segments	Effective segmentation	Small dataset, segmentation errors	Moderate
Cheng et al. (2015)	BoW + SVM	3064 MRI images	Tumor region augmentation for better classification	Simple and interpretable	Feature extraction limitations	High
Ismael et al. (2018)	2D DWT + Gabor Filter + Neural Network	3064 MRI images	91.9% accuracy	Good texture analysis	Lower accuracy than deep learning methods	Moderate
Tahir et al. (2019)	Daubechies Wavelet + SVM	3064 MRI images	86% accuracy	Effective wavelet-based approach	Feature extraction constraints	Moderate
Paul et al. (2017)	CNN	989 axial MRI images	Achieved 90.26% accuracy	Strong feature learning	Small dataset, lacks segmentation	High
Afshar et al. (2018)	Capsule Network (CapsNet)	3064 MRI images	More robust to spatial variations	Handles rotation better than CNNs	Lower accuracy (86.56%), requires large training data	Very High
Afshar et al. (2019)	CapsNet with Bounding Box	3064 MRI images	90.89% accuracy, improved over prior CapsNet	Improves localization of tumors	High computational cost	Very High
Zhou et al. (2018)	DenseNet + LSTM	989 MRI images	92.13% accuracy	Good sequence modeling	Requires more data	High
Pashaei et al. (2018)	CNN + Extreme Learning Machine (ELM)	3064 MRI images	93.68% accuracy	Fast training	Lacks segmentation	High
Abiwinanda et al. (2018)	CNN	2100 MRI images	84.19% accuracy	Simple CNN-based approach	Lower than ensemble models	High
Ghassemi et al. (2020)	GAN + CNN	3064 MRI images	Improved data augmentation	Generates synthetic data for training	Computationally expensive	Very High
Guo et al. (2019)	Graph CNN for PET	ADNI dataset (Alzheimer’s)	93% accuracy (2-class), 77% (3-class)	Effective for brain mapping	Limited to Alzheimer’s	High
Anaraki et al. (2019)	Genetic Algorithm + CNN	3064 MRI images	94.2% accuracy	Adaptive feature selection	GA-based tuning is complex	Very High
Ayadi et al. (2021)	Deep CNN	3064 MRI images	Improved classification	Strong classification accuracy	High training time	High
Deepak & Ameer (2019)	CNN + Transfer Learning	3064 MRI images	97.10% accuracy	Pre-trained model improves performance	May not generalize to unseen data	Moderate
Sejuti & Islam (2021)	CNN + SVM	3064 MRI images	97.10% accuracy	Hybrid model improves classification	Feature extraction dependency	High
Kumar et al. (2021)	ResNet50 + Global Average Pooling	3064 MRI images	97.48% accuracy	Deep residual learning	Risk of overfitting	High
Kakarla et al. (2021)	CNN with 8 pooling layers	3064 MRI images	97.42% accuracy	Effective pooling strategy	Parameter tuning required	High
Guan et al. (2021)	CNN + Bounding Box	3064 MRI images	98.04% accuracy	Optimized for efficiency	May not handle small-scale tumors well	Very High

## Proposed methodology

**Fig. 1:**
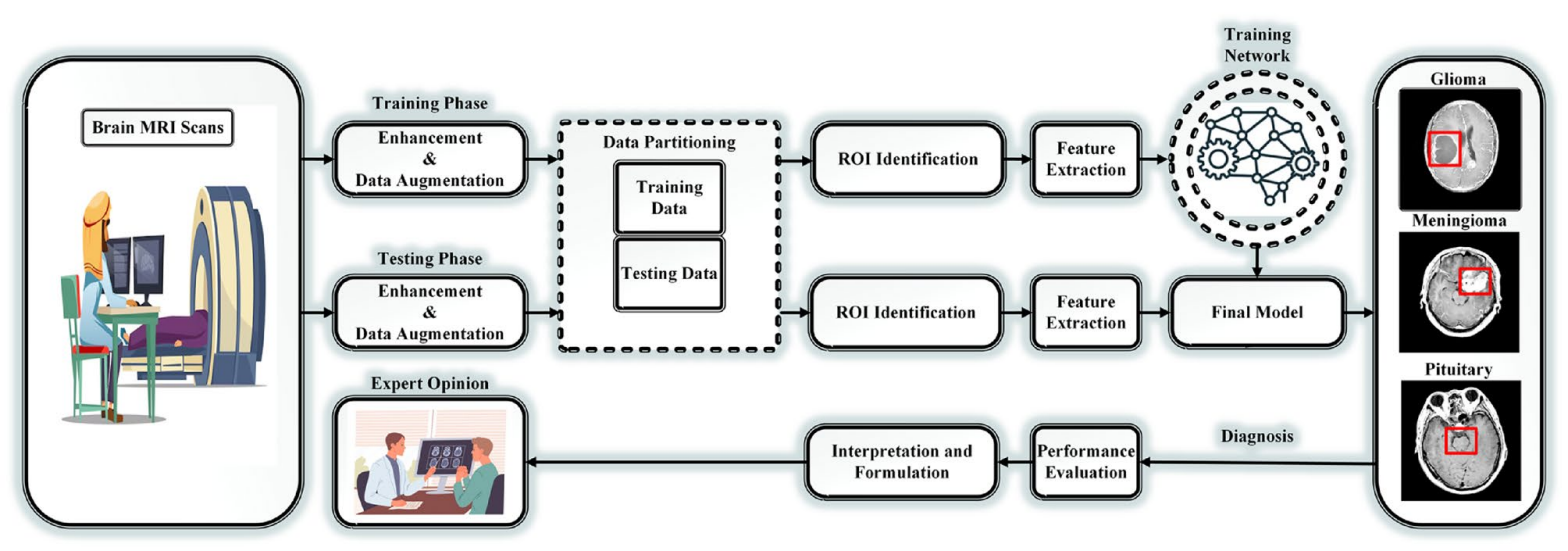
The block diagram of the critical stages involved in the classification of brain tumors.

**Fig. 2:**
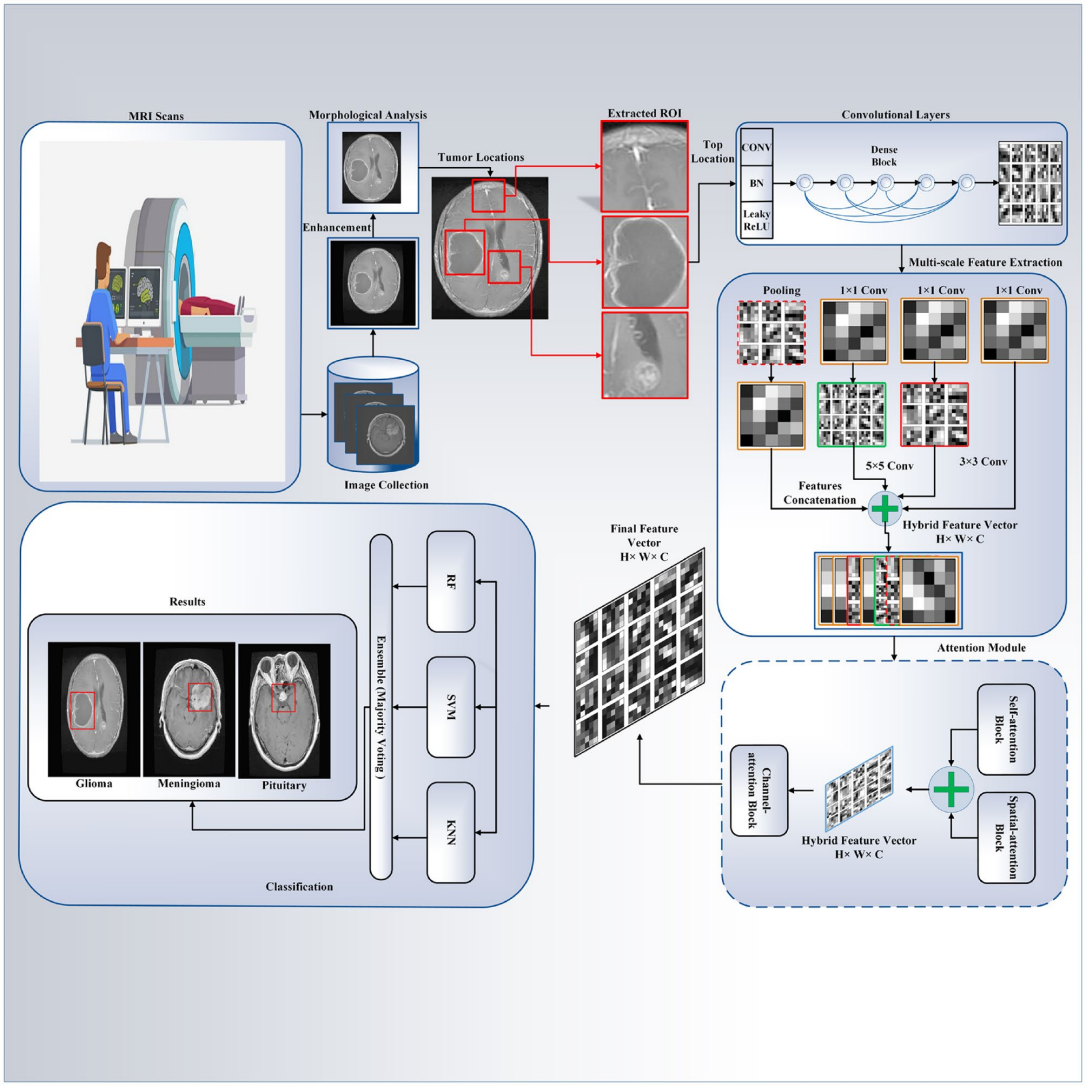
The sequential working mechanism of the proposed model.

A comprehensive overview of the proposed technique is provided in Fig. 1, which highlights the critical stages and components of BTC. The sequential working mechanism of the proposed model is illustrated in Fig. 2, which demonstrates the interaction of components to generate the final result. The proposed strategy improves classification accuracy by expanding the potential for image enhancement, data augmentation, segmentation, and feature extraction.

The critical phases of the proposed model that are necessary for BTC are summarized as follows:

### Enhancement and morphing analysis

To enhance the quality of brain tumor images, a preprocessing technique is used, which combines both guiding filtering and AGSW methods^48^. The goal is to improve the visibility of the important features in the images and to reduce the noise to help the overall detection process. Their gradients are first obtained to estimate the local structure in the tumor images. Then, using the gradients as supplementary information, the guidance image is filtered using AGSW. This filtering process improves the quality of the guidance image and preserves its edges and intricate details. The guided filter uses this improved guidance image to improve visibility in the original brain tumor images. Generally, before using reference images, they need to be resized and converted to appropriate color spaces or representations to guarantee their compatibility and alignment with the target image. To achieve better visibility, the goal of restoring or enhancing the target image must align with the selection of reference images. For example, the gradients of the local structure in the tumor image are highlighted in the reference image to improve the visibility of specific structures within the image. A clean variant of the identical image can be utilized as a suitable reference to reduce noise. The relationship between the guided image $Y_{i}$ and the filtering output $Z_{i}$ is governed by a local linear model. In the window $N_{k, \theta }$ it is specified that $Z_{i}$ is a linear transformation of $Y_{i}$. Equation (1) represents the filtered output:

1 $$\begin{aligned} Z_i=a_k Y_i+b_k ; \quad n \in N_{k, \theta } \end{aligned}$$

In this context, $N_{k, \theta }$ denotes the window, while $a_k$ and $b_k$ represent the gain and deviation coefficients, respectively. To acquire accurate edge information, the anisotropic Gaussian weighting factor is converted into the square error loss function $E(a_{a},b_{k})$ of the guided filter, as denoted by Equation (2). Subsequently, the error between *O* and the input image *I* is reduced to obtain the optimal parameters.

2 $$\begin{aligned} E\left( a_k, b_k\right) =\sum _{i \in N_{k, \theta }} w_{i . \theta }\left( a_k Y_i+b_k+a_k-p_i\right) ^2+\varepsilon a_k^2 \end{aligned}$$

The weight of pixel *i* in window $N_{k, \theta }$ is represented by the symbol $w_{i, \theta }$. The regularization parameter is indicated as $\varepsilon$. The gain coefficient is denoted as $a_k$. The input image from the dataset is represented as $P_i$. The guided image is designated as $Y_i$. In order to obtain accurate and exact values for the parameters $\overline{a_i}$ and $\overline{b_i}$, as shown in Equation (3), the relevant weighting operations $G_{ij}$ and $G_i$ are performed:

3 $$\begin{aligned} \begin{aligned}&\overline{a_i}=\frac{1}{\xi } \sum _{k \in N_{i, \theta }} w_{k, \theta } a_k \\&\overline{b_i}=\frac{1}{\xi } \sum _{k \in N_{i, \theta }} w_{k, \theta } b_k \end{aligned} \end{aligned}$$

The term $w_{k, \theta }$ represents the weight assigned to the pixel *k* within the window $N_{k, \theta }$. The weighted sum is represented by the symbol $\xi$, while $a_k$ and $b_k$ are used to denote the gain coefficients. The output processed by the filter, represented as Equation (4), is obtained by computing the values of $\overline{a_i}$ and $\overline{b_i}$ for each individual pixel:

4 $$\begin{aligned} Z_i=\overline{a_i} Y_i+\overline{b_i} \end{aligned}$$

**Fig. 3:**
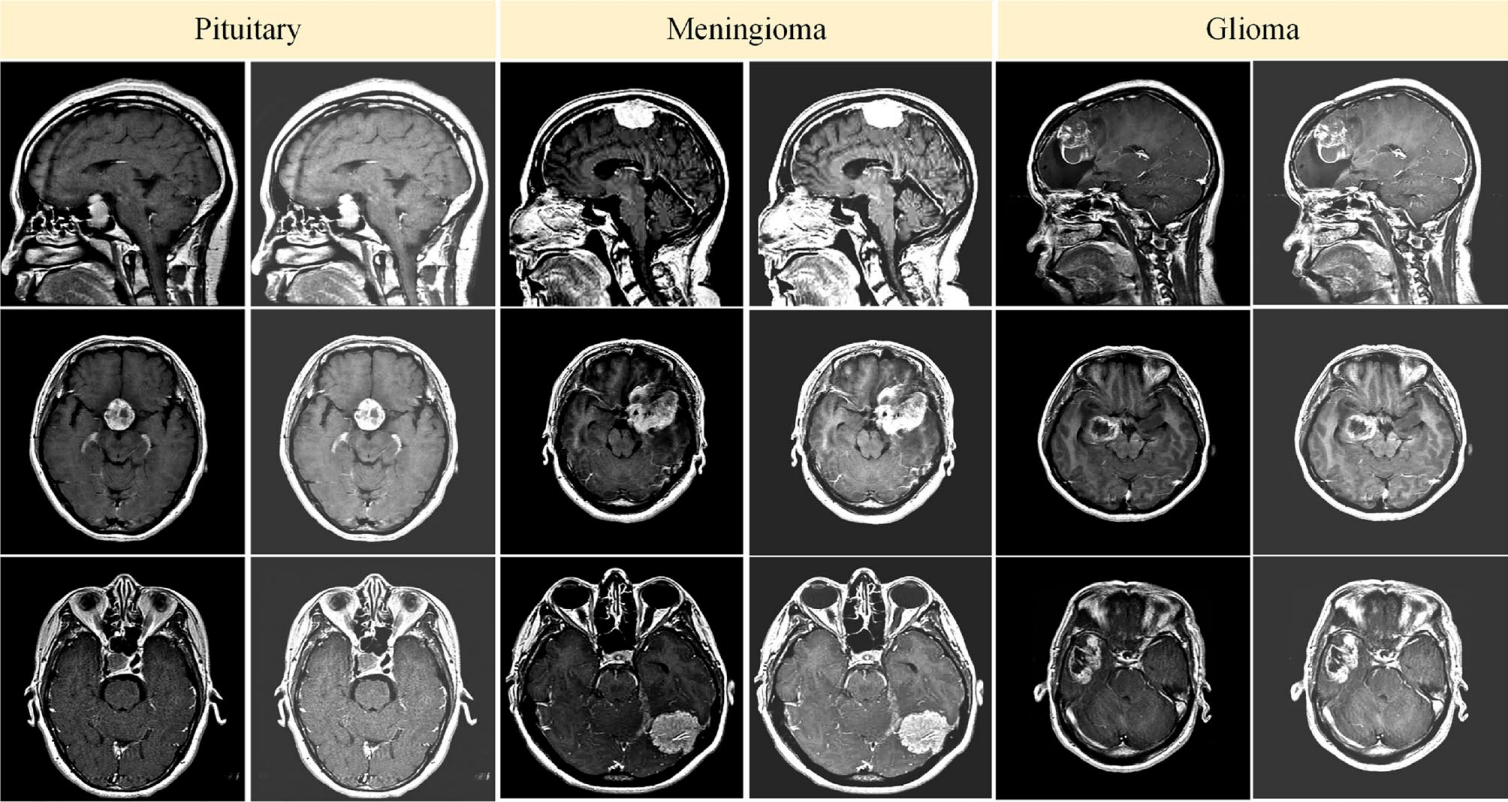
MRI images prior to and following the enhancement operation.

In this particular situation, $Z_i$ represents the output of the filtering process, $Y_i$ represents the guided image, and $\overline{a_i}$ and $\overline{b_i}$ signify the steady linear parameters of the gain coefficient. The effect of the improvement is evident in Fig. 3.

**Fig. 4:**
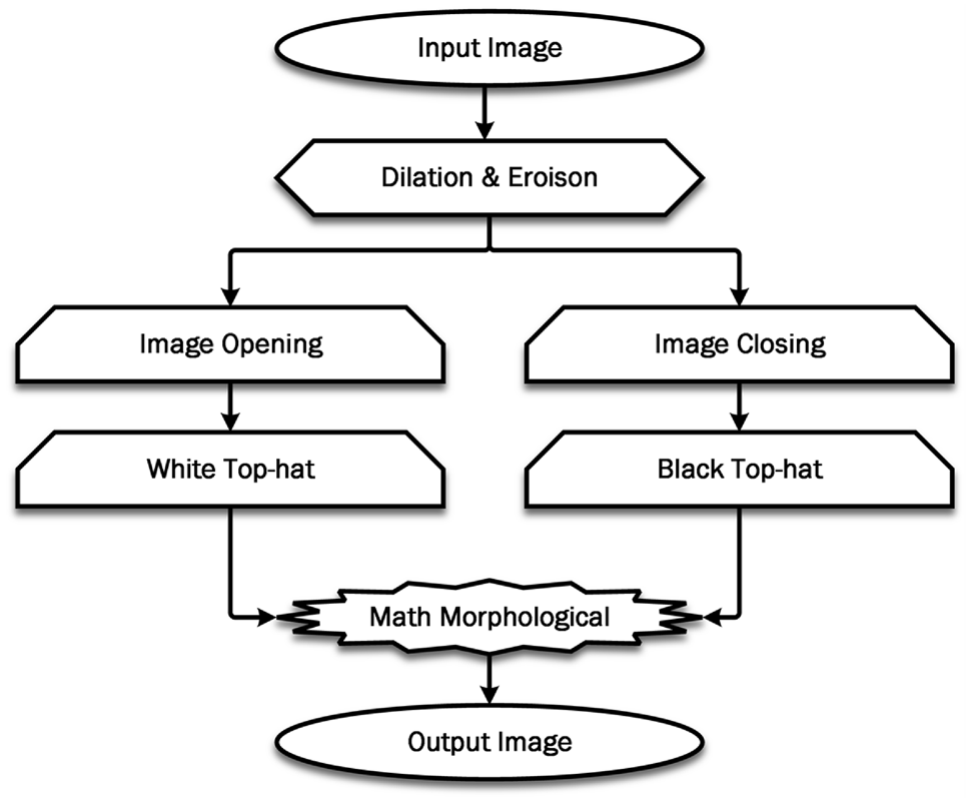
The steps involved in morphological process.

Before identifying the precise position of the tumor in enhanced images,it is necessary to remove non-tumor regions to ensure accurate and reliable results^20^. The structuring element (*SE*) is utilized to acquire the required image structures. The value of each pixel is determined by the surrounding pixels and the corresponding value in the input. The resulting image generated by this morphological technique maintains the dimensions of the augmented image. Fig. 4 elucidates the methodology of the morphological operation.

### ROIs generation

After improvement, the brain tumor MRI image must be divided into segments. The goal is to extract ROIs of high-quality from the tumor image to improve classification accuracy. For this purpose, the U-Net architecture is implemented. The remarkable performance of the various U-Net arbitrators has been seen in the field of medical segmentation^49^. For this experiment, the U-Net encoder used is SE-ResNet101. In this 101-layer residual network, Squeeze replaces the residual blocks and extinction blocks through integration. By including SE blocks, the parameter can be added to each channel of the convolutional block, allowing for the adjustment of the weights assigned to every feature map in the network.

**Fig. 5:**
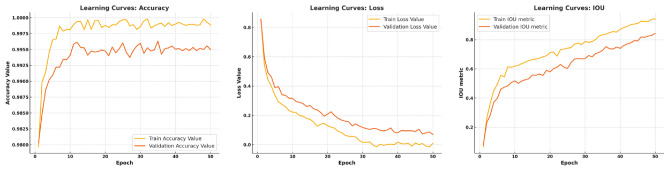
Accuracy of the segmentation method during training and testing.

**Fig. 6:**
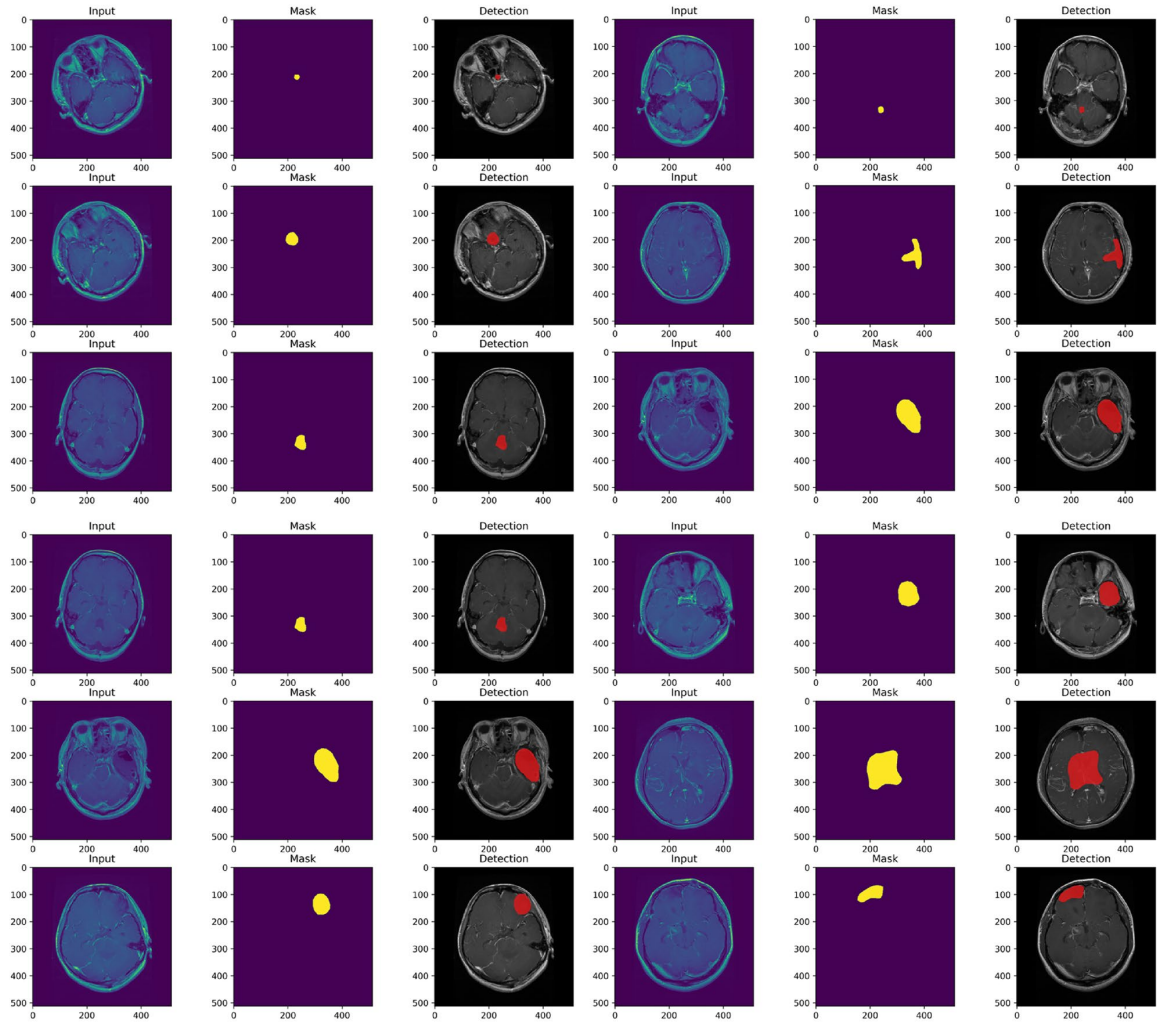
The model’s segmentation results.

Global pooling on each individual channel is implemented to obtain a comprehensive understanding by combining feature maps into a single numerical vector. These techniques yield a vector of size *n*, where *n* represents the convolutional channels *C*. This vector was fed into a two-layer neural network comprising fully connected (FC) layers with Rectified Linear Unit (ReLu) activation and Sigmoid function. This produced a vector of similar dimensions that was used as a weight in the original feature maps. Each channel is assigned a scale based on its level of importance in this complete approach. The dataset was partitioned into two sets for analysis: training sets and test sets. The training sets comprise 70% of the dataset and the test set comprises 30%. Empirical methods determined the two model hyperparameters: the batch size was fixed at 4, and the number of epochs was set at 60. Fig. 5 shows the results of segmenting brain tumors following the training and testing process. Conversely, Fig. 6 shows the projected tumors.

### Feature extraction

DenseNet was chosen for feature extraction due to its proficiency in handling complex features, making it suitable for BTC task. In contrast to traditional CNN, DenseNet creates direct connections among layers, facilitating feature reuse and ensuring a consistent gradient flow. This design emphasizes intricate features in MRI scans, minimizing redundancy and improving efficiency and accuracy. DenseNet collects more comprehensive data while using fewer parameters than deeper networks such as ResNet, which face challenges in medical imaging due to the variability in tumor size, shape, and intensity.

Before the finalization of DenseNet, a thorough assessment was performed comparing it with leading architectures, including ResNet, InceptionNet, MobileNetV2 and Inception-ResNet. Each model possesses different advantages: MobileNetV2 is particularly strong in its lightweight architecture, while Inception-based networks demonstrate effectiveness in extracting features across multiple scales. However, DenseNet reliably exhibited higher accuracy tailored to our specific requirements. Ensemble classification techniques and attention mechanisms were integrated into DenseNet to improve performance. The effectiveness of this combination in diagnosing brain cancer led to a notable improvement in patient outcomes. The extensive trials validated our conclusions, affirming the reliability and precision of the proposed approach in real-world clinical applications.

DenseNet processes segmented images to accurately identify brain tumors. The architecture effectively addresses the issue of gradient vanishing by minimizing the gap between the input and output^50^. The network streamlines data representation across various levels by providing a map format with reduced features. This minimizes the risk of overfitting, maintains data integrity during transmission, and improves CNN understanding when dealing with limited datasets. Regularization techniques and the loss function method supervise each layer, effectively minimizing overfitting by decreasing connections between layers and facilitating a more manageable training process. DenseNet consists of three primary components: the dense block, the transition layer, and the growth rate. The structure comprises *O* dense units organized into groups, where each dense block encompasses *N* stages. The feedforward methodology facilitates the establishment of connections between each step and the subsequent stages within a dense block, ultimately producing the result $B_n$ after completing the dense section. Equation (5) provides a mathematical representation of the $n^{th}$ layer.

5 $$\begin{aligned} B_n = I_n([B_1,B_2,..........B_{n-1}]) \end{aligned}$$

This variable describes the concurrent measurement technique and fusion process that is currently taking place. To further decrease the size of the feature maps between each dense segment, a transition layer is introduced, which consists of a $1 \times 1$ convolutional layer followed by an $2 \times 2$ average pooling layer. The results of each stage are combined to create a final feature map. The dimension of the layers at the location *N* is defined by a function that incorporates the growth rate. This is defined as: $g(n-1) + g_0$, where $g_0$ is the number of sections in the original input image. To improve the effectiveness of the variable and maintain authority over the entire network, the size of the growth rate *H *size *g* is limited. This could aid in the preparation for dealing with overfitting or the complexity of the model. The growth rate parameter regulates the amount of fresh data or information added to each network layer or stage.

#### Multiscale feature maps

**Fig. 7:**
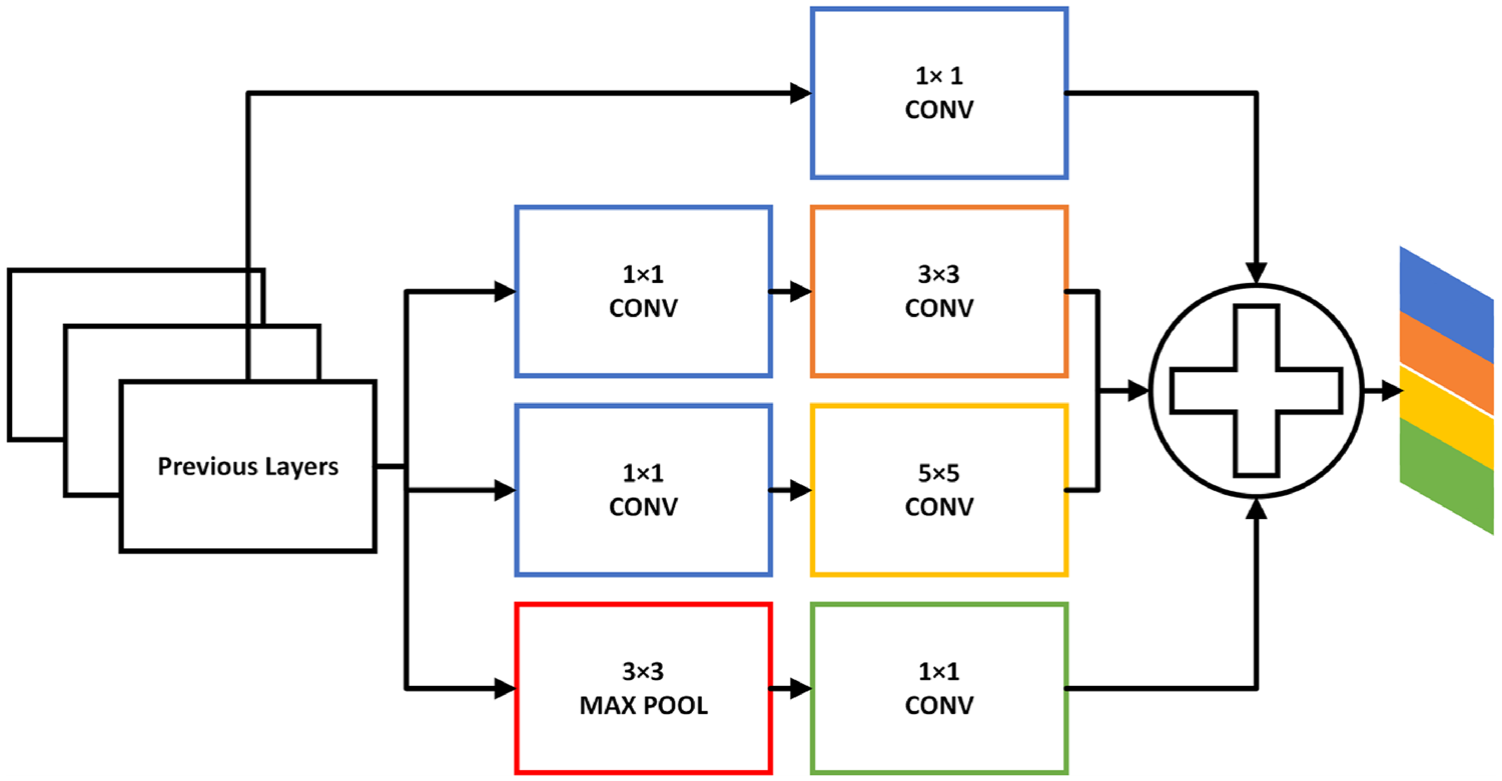
The process of multiscale feature extraction.

Due to the concatenation of features from one layer to another, DenseNet can produce duplicate data. With regard to the execution of numerous computations, the network exhibits exceptional efficiency. A multiscale strategy is implemented to address this problem. In contrast to conventional DenseNet one-scale convolutional kernels, the multiscale assisted convolutional kernels effectively manage anomalies and streamline intricate data processing. As a result, Fig. 7 illustrates the GoogleNet Inception-based multiscale feature extraction model used in this study. This model can simultaneously input images and convolving filters of varying sizes^51^. Concatenate the convolution output into the channel dimension to obtain the multiscale feature map. By decreasing the scale of feature maps before applying a filter of greater magnitude and utilizing the pooling technique for spatial downsampling, the capability of representing the network is further enhanced. After aggregating data from different branches, the low- and high-level characteristics were documented. Using this network, more comprehensive visual representations of the contextual intricacies present in brain tumor MRI images at different magnifications have been acquired.

Discerning the more salient features is critical for a complete understanding of brain MRI images. Implementing an attention module enables the acquisition of more discriminative attributes while ignoring extraneous input. The spatial attention block extracts interdependencies between features at any position, whereas the self-attention block extracts interdependencies between features along spatial locations. To generate a comprehensive spatial relationship in MRI images, the refined feature map is formed by combining the two attention maps^3^.

#### Self-attention block

To fully capture spatial dependencies, the input image feature is initially reshaped into $\mathbb {R}^{Y \times D}$, where $Y=H \times W$ and *D* represent the channel length and spatial domains, respectively. This transformation is denoted as $F_{i j}=\left[ f_{i j}^1, f_{i j}^2, f_{i j}^3, \ldots , f_{i j}^D\right] \in R^{H i \times W j \times D}$. We assigned the input features to the three heads *Q*, *K*, and *V*. The calculations are as follows:

6 $$\begin{aligned} & Q_i=F W_i^Q+e^Q \end{aligned}$$

7 $$\begin{aligned} & K_i=F W_i^K+e^K \end{aligned}$$

8 $$\begin{aligned} & V_i=F W_i^V+e^V \end{aligned}$$

where $W_i^Q\in R^{D \times C_{k}}$, $W_i^K\in R^{D \times C_{k}}$, and $W_i^Q\in R^{V \times C_{k}}$ are the parameter matrices. The letters $C_k$, $D_k$, and $C_k$ represent the dimensions of *Q*, *K*, and *V* in each cranium, respectively, while the deviation terms are denoted as $e^{Q}\in R^{C_{k}}$, $e^{K}\in R^{C_{k}}$, and $e^{V}\in R^{C_{k}}$. The subsequent equation may be used to determine the spatial attention map for each head:

9 $$\begin{aligned} \text{ head } _i=\operatorname {Attention}\left( Q_i, K_i, V_i\right) =\operatorname {Softmax}\left( \frac{Q_i, K_{i i}^T}{C_k}\right) V_i \end{aligned}$$

where $head_{i} \in R^{N \times C_{v}}$. Following this, concatenation and addition operations are used to obtain the final self-attention features:

10 $$\begin{aligned} F_{SA}=(Q,K,V)=Concat(head_1, head_2,........head_h)W^\sigma +b^\sigma \end{aligned}$$

In the given context, the projection matrix $W^\sigma \in R^{Y \times D}$, the bias term $b^\sigma \in R^D$, and multi-head attention are denoted as $M(\cdot )$.

#### Spatial attention block

To extract the interdependencies of features along spatial positions, a spatial attention block is implemented due to the considerable variation in size and shape of brain lesions. This segment evaluates the importance attributed to each spatial location on the feature map. Assume that the input feature map for the network is represented as follows: $F\in R^{H \times W \times D}$. Initially, cross-channel average pooling (*CAP*) and cross-channel maximum pooling (*CMP*) are executed concurrently in this block to construct feature maps $F_1\in R^{H \times W \times 1}$ and $F_2\in R^{H \times W \times 1}$, correspondingly. The output $F^{\prime } \in R^{H \times W \times 2}$ is obtained by combining the values of $F_1$ and $F_2$, is generated using the ReLU activation function. This output can be mathematically represented as:

11 $$\begin{aligned} F^{\prime }=\psi (\operatorname {concat}(C A P(F), C M P(F))) \end{aligned}$$

Following activation of the intermediate output $F^{\prime }$ with the sigmoid function and a convolutional layer of size $1 \times 1$, a spatial attention map $F^{\prime \prime } \in R^{H \times W \times 1}$ is generated. $F_{sab} \in R^{H \times W \times D}$ is the result of element-wise multiplication between $F^{\prime \prime }$ and *F*; the result is denoted as:

12 $$\begin{aligned} F_{s a b}=\sigma \left( \phi ^{1,1,1}\left( F^{\prime }\right) \right) \odot F \end{aligned}$$

Where $\sigma$ is the sigmoid activation and $\odot$ is the element-wise multiplication operation.

#### Aggregation

The network integrates the attention maps produced by the spatial attention block (SAB) and self-attention block (SB) to build a polished feature map named $F_{agg} \in R^{H \times W \times C}$. The feature map precisely captures the spatial correlation observed in MR images:

13 $$\begin{aligned} F_{agg} = F_{SAB} \oplus F_{SB} \end{aligned}$$

### Channel attention block (CAB)

Channel-wise dependencies are identified during the process of traversing irrelevant channels, as class-specific characteristics may have been lost in the aggregated feature map. In order to determine the importance of individual channels in the feature map $F_{agg} \in R^{H \times W \times C}$, channel-wise attention weights $F^{*} \in R^{1 \times 1 \times C}$ are generated via two $1 \times 1$ conv layers with ReLU and sigmoid activation and global max pooling (GMP). Using element-wise multiplication in conjunction with $F_{agg}$, the following computations produce the final channel-wise attention feature maps:

14 $$\begin{aligned} F_{C A B}=\sigma \left( \phi ^{1,1, C}\left( \psi \left( \phi 1,1, \hat{C}\left( \operatorname {GMP}\left( F_{\text{ agg } }\right) \right) \right) \right) \right) \odot F_{\text{ agg } } \end{aligned}$$

The symbols $\sigma$ and $\psi$ denote the ReLU and sigmoid activations, respectively. The constant $\hat{C}=C / 8$ is a value that was determined through empirical calculation.

### Classification

Ensemble models have experienced notable improvements in accuracy and effectiveness when applied to classification tasks^52^. These models have become increasingly popular in recent times. In this investigation, brain lesions are diagnosed using ensemble learning techniques, as the highest degree of precision is achieved by integrating numerous classifiers. In order to partition brain tumors detected on MRI scans into three discrete categories, the suggested methodology employed a voting classifier that soft-voted on the RF, SVM, and KNN models. The ultimate result is determined by the class that accumulates the most votes. The subsequent section explains the operation of the ensemble model.

15 $$\begin{aligned} \hat{p}=\sum _i^n R F_i, \sum _i^n S V M_i, \sum _i^n K N N_i \end{aligned}$$

Equation (15) produces the forecast probabilities for each test sample. The probabilities are further evaluated using the soft voting criterion, and the probabilities for each test case are calculated using RF, SVM, and KNN. Using the dataset comprising brain tumor MRI images, the efficacy of the proposed model is assessed in two ways. Each available feature of a brain tumor is utilized in the detection procedure to filter it. The second step is to acquire convolutional features for machine learning models via preprocessing imagery.  In order to assess the performance of the proposed model, we have employed 5-fold CV, which is a standard method in machine learning. In this, the data is divided into five equal subsets. In each run, four subsets are used for model training, and the remaining subset for testing. By doing so, each sample is tested only once, and the model is tested on unseen data repeatedly. By calculating the outcome over all five folds, we have a better and more balanced estimation of the accuracy and generalization of the model outside of training data^53^.

## Evaluation and results

### Dataset

**Fig. 8:**
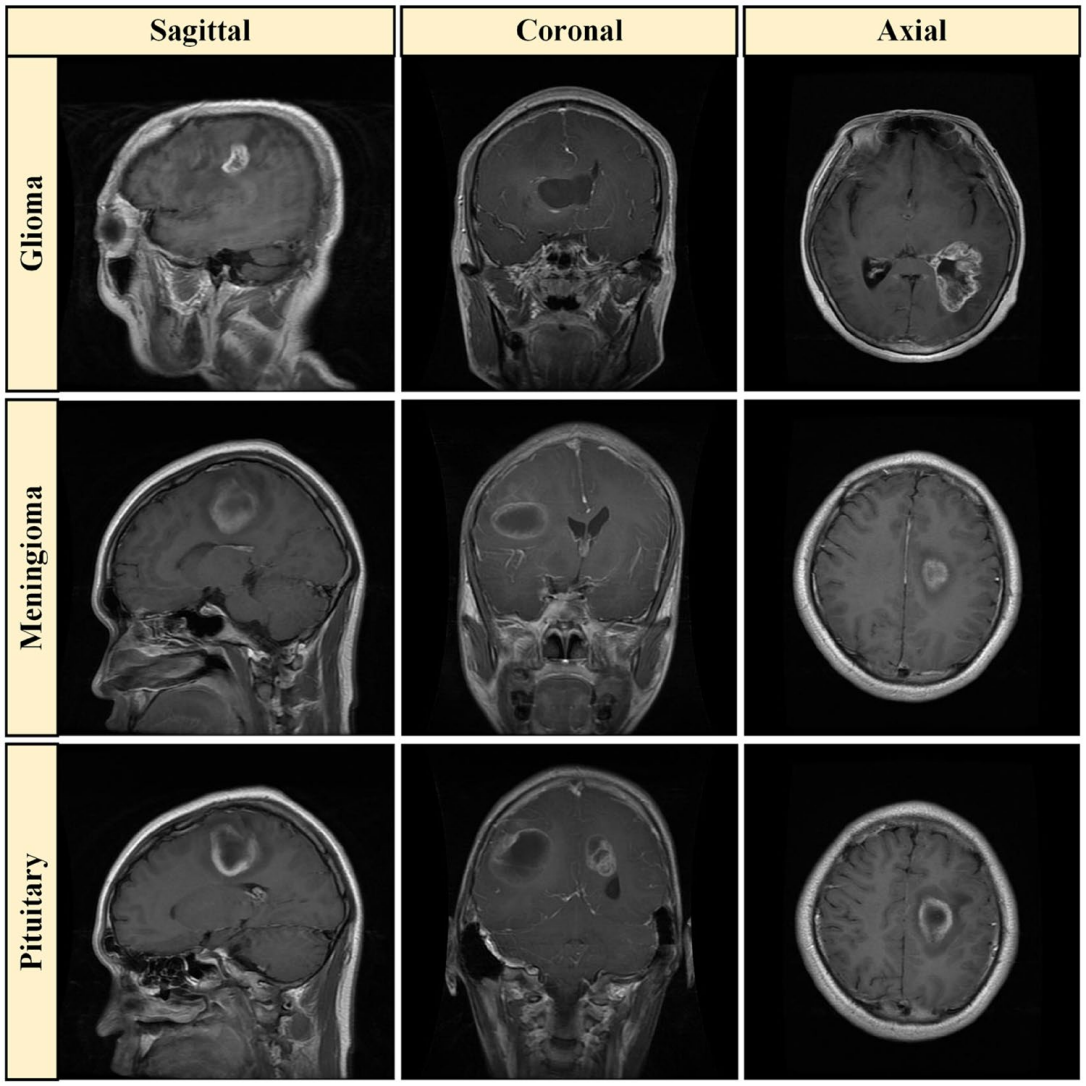
Illustration of the sample brain tumor images.

The effectiveness of the proposed model was assessed using the publicly accessible brain tumor dataset that was initially provided by Cheng et al.^30^. This dataset comprises 3064 T1-weighted enhanced contrast brain MRI scans with a voxel spacing of and a resolution of pixels per image. Data were collected from 233 patients treated in two state institutions in Guangzhou and Tianjin, China, between 2005 and 2010. The collection includes 930, 708, and 1426 occurrences of brain malignancies in the Pituitary, Meningioma, and Glioma, respectively, in axial, coronal, and sagittal views. In addition to a comprehensive description of the dataset, tumor masks, tumor class labels, tumor borders, and patient IDs, the dataset is available in the MATLAB format (.mat). In general, the visual quality of MRI images is improved by preprocessing them to enhance their brightness and contrast^54^. These images may contain artifacts and inconsistencies in intensity levels as a result of the use of various imaging modalities. Consequently, they require cleansing and enhancement to enhance their contrast value. Table 2 provides a comprehensive description of the dataset, while Fig. 8 illustrates preprocessed samples of the three categories of brain tumors. The primary objective is to enhance the visual quality of the photographs by expanding the dynamic range of grayscale values.

**Table 2:** Summary of MRI images and participant distribution for each tumor group.

Tumor’s group	No. participants in each group	No. MRI in each group	MRI images in various planes/views
Glioma	89	1426	Coronal: 437, sagittal: 495, transverse (axial): 494
Meningioma	82	708	Coronal: 268, aagittal: 231, transverse (axial): 209
Pituitary	62	930	Coronal: 319, sagittal: 320, transverse (axial): 291
Overall	233	3064	Coronal: 1024, sagittal: 1046, transverse (axial): 994

### Preprocessing and data augmentation

**Fig. 9:**
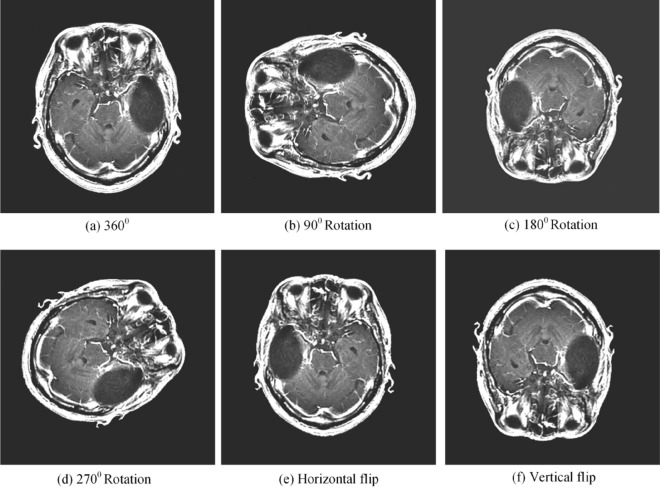
The results obtained using data augmentation techniques.

 Normalization techniques are widely used to improve the quality of input data used for classification and ensure consistent and reliable network convergence^55^. The convolutional kernel is a technique that can be used to assess pixel intensity in brain MRI images. Regrettably, the efficacy of this approach is contingent upon the brightness of the pixels. Normalizing the data is essential prior to performing network optimization procedures, regardless of the existence of substantial participant differences and data collection conditions, due to the substantial variation in values between and within individuals. The Min-Max method significantly improved the network training process by rescaling the intensity values of the input images to a range of 0 to 1. In addition, contrast enhancement techniques were implemented to enhance the visual clarity and distinctiveness of the MRI images, which can be altered by incorrect intensity levels and defects. The quantity of the dataset was increased using data augmentation techniques, including image rotation at varying angles and vertical and horizontal flipping. These augmentations were applied to reduce overfitting and improve the model’s generalization by effectively expanding the sample size. As a result, the dataset has quadrupled in size to contain 12,256 sample images at present. The efficacy of data enhancement procedures is illustrated in Fig. 9, and additional details regarding contrast enhancing techniques are offered in “Enhancement and morphing analysis” section.

**Table 3:** Exploring optimization techniques and learning rates for improved accuracy.

Optimization algorithms	0.1	0.01	0.001	0.002	0.003	0.004
Adam	81.82	83.11	84.79	85.46	86.27	88.72
SGD	87.83	93.62	89.22	91.77	91.06	92.43
Adadelta	89.02	85.87	81.31	82.18	83.69	83.85
RMSprop	82.79	84.88	80.52	81.17	83.47	81.14
Adagrad	84.88	92.85	98.00	97.33	99.67	98.56

**Table 4:** Measures of average accuracy for the proposed system at various epochs.

Number of epochs	50	100	150	200
Overall accuracy	99.21	99.67	98.97	99.09

**Table 5:** Exploring average accuracy of proposed system at different dropout rates.

Dropout rates	0.1	0.3	0.5	0.7
Overall accuracy (%)	99.05	99.10	99.67	98.98

### Competitors

The reliability and utility of the proposed BTC approach were evaluated compared to a variety of cutting-edge methods^20,21,30–47^. The results of the experiments indicate that the proposed method is highly effective.

### Evaluation matrix

A variety of evaluations were implemented, including the F1-score, sensitivity, precision, specificity, and precision of the proposed model, to verify its functionality. Four measures were used to evaluate the predicted classes: false positives (FP), false negatives (TN), true positives (TP), and false negatives (FN). The mathematical representation of each of these numbers is as follows:

16 $$\begin{aligned} & \text{ accuracy } =\frac{T P_N+T N_N}{T P_N+T N_N+F P_N+F N_N} \end{aligned}$$

17 $$\begin{aligned} & \text{ specificity } =\frac{T N_N}{T N_N+F P_N} \end{aligned}$$

18 $$\begin{aligned} & \text{ sensitivity } \text{(recall) } =\frac{T P_N}{T P_N+F N_N} \end{aligned}$$

19 $$\begin{aligned} & \text{ precison } =\frac{T P_N}{T P_N+F P_N} \end{aligned}$$

20 $$\begin{aligned} & f_1- \text{ score } =2 \times \frac{ \text{ recall } \times \text{ precision } }{ \text{ recall } + \text{ precision } } \end{aligned}$$

### Hyperparameters

To improve the quality of input images, normalization and enhancement techniques are implemented, while data augmentation techniques are used to facilitate the training process. Instead of taking a constant train-test split, we have used 5-fold CV for better estimation of the model. This process distributed the dataset into five subsets of nearly equal size. With each fold, approximately 80% of the data was assigned for training and 20% was used for testing. With this setup, every sample appeared once as test data and four times as training data. By averaging across all five folds, we could estimate the model’s classification performance more accurately, with better balance, higher accuracy, and greater reliability. The training sets within all the folds were also used to carry out experiments for selecting the optimal hyperparameters to employ for the final model. The results are presented in Tables 3, 4, and  5. The model exhibits high accuracy with a batch size of 32, a dropout rate of 0.5, 100 epochs, and a learning rate of 0.003. The results are presented in Tables 7 and  8, and we employ a 5-fold CV according to the method outlined by Cheng et al.^30^ to evaluate the effectiveness of the proposed model. In general, the proposed model significantly mitigates overfitting, converges more rapidly, and offers precise retrieval capabilities with minimal processing power. It is a straightforward and user-friendly approach that can help radiologists determine the appropriate classification for an object. Ultimately, our proposed approach is a robust framework that is effective for brain sorting tasks.

### Evaluation and results

**Fig. 10:**
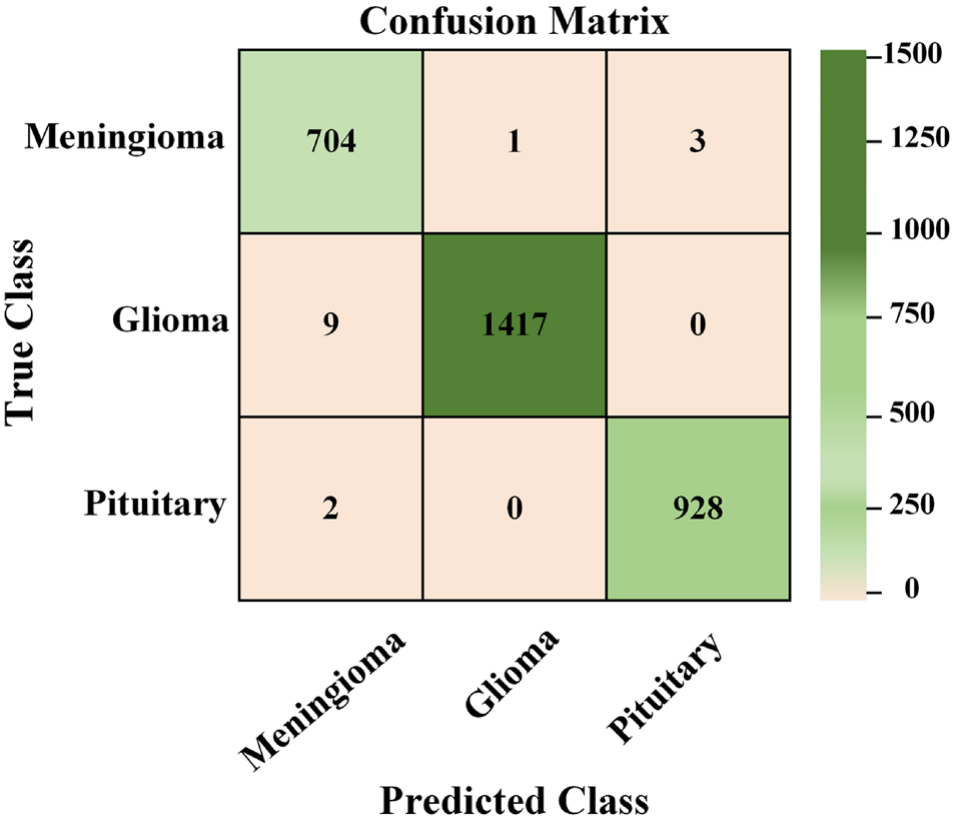
The confusion matrix of the proposed model.

To evaluate the effectiveness of the proposed method, we constructed a confusion matrix that considers both correct and incorrect predictions of the model. The confusion matrix presented in Table 6 and Fig. 10 shows that the proposed model correctly classified 3049 samples and only 15 cases were misclassified. This yields an overall accuracy of 99.67%. Significantly, the most accurate predictions for Glioma were obtained, mainly due to the extensive training dataset created through many augmentation procedures. The inclusion of a well-balanced dataset resulted in a notable improvement in classification accuracy. The performance of the classifier was evaluated in terms of accuracy, sensitivity (recall), specificity, precision, and F1-score for each category of tumor using the confusion matrix previously mentioned. The performance of the classifier for each brain tumor category is illustrated in Table 7. It is important to note that the confusion matrix presented in Table 6 corresponds to a single representative fold from the 5-fold CV performed on the original dataset of 3,064 images, yielding a fold-specific accuracy of approximately 99.51%. The class-wise metrics shown in Table 7 are also derived from this same fold, and their macro-average accuracy is 99.67%, which coincides with the average overall accuracy obtained from the complete 5-fold evaluation using the augmented dataset. Presenting both perspectives provides clearer insight into the model’s performance: Table 6 illustrates its behavior in one specific run, while Table 7 highlights its consistency and robustness across the entire dataset. The proposed technique demonstrated extraordinary performance in the areas of precision, sensitivity, specificity, accuracy, and F1-score for Pituitary, Glioma, and Meningioma tumors. The proposed model exhibited a sensitivity (recall) of 0.9944% for Meningioma, 0.9937% for Glioma and 0.9978% for Pituitary, as well as specificities of 0.9953%, 0.9994% and 0.9986%, respectively. In addition, it had an accuracy of 0.9951% for Meningioma, 0.9967% for Glioma, and 0.9984% for Pituitary. Our technique is highly beneficial for the accurate diagnosis of brain lesions using MRI data, as evidenced by the exceptional precision of the model and F1-score values.

**Table 6:** Confusion matrix showing predicted and actual values for each class.

Actual values	Meningioma	Glioma	Pituitary
Meningioma	704	1	3
Glioma	9	1417	0
Pituitary	2	0	928

As a result of these findings, the proposed method for classifying malignancies in sample images is highly effective. Notable is that our method obtained high specificity values for all classes, indicating an accurate diagnosis of sample images that do not contain the condition in question. Our approach shows greater efficiency and performance compared to previous methods. The increase in the number of sample images improved the efficiency of the model while resolving the issue of overfitting. The proposed method eliminates the need for manual segmentation and does not require prior knowledge of the to-be-retrieved feature types, which impacts the generalization ability of the network. The results led us to the conclusion that our model is highly generalizable and stable. In addition, the proposed method is suitable for several applications, including the classification of breast cancer.

**Table 7:** Evaluation of proposed methodology using various quality measures.

Tumor type	Accuracy	Sensitivity (recall)	Specificity	Precision	$F_1$-score
Meningioma	0.9951	0.9944	0.9953	0.9846	0.9895
Glioma	0.9967	0.9937	0.9994	0.9993	0.9965
Pituitary	0.9984	0.9978	0.9986	0.9968	0.9973
Overall accuracy	0.9967				

To perform a thorough assessment, the proposed model is compared with several prominent deep learning architectures, such as ResNet50, Inception-ResNet, MobileNet, InceptionNet and GoogLeNet. Table 8 demonstrates the impressive accuracy of the proposed model i.e, 99.67%, surpassing that of GoogLeNet (99.15%), Inception-ResNet (98.89%), MobileNet (98.72%), InceptionNet (98.55%), and ResNet50 (98.39%). However, precision alone cannot fully convey the complete story. The model demonstrated outstanding performance on several key metrics, achieving a F1-score of 99.60%, specificity of 99.64%, precision of 99.35%, and sensitivity of 99.60%, leading to a balanced and highly reliable classification performance. This degree of accuracy and uniformity results from meticulously organized improvements. We improved image preprocessing, refined segmentation with U-Net and SE-ResNet101, extracted features on various scales, and implemented attention mechanisms to emphasize the most significant tumor characteristics. To improve overall classification accuracy, we employed an ensemble learning approach that integrates RF, SVM, and KNN, enabling the model to generate more reliable and insightful predictions. Our approach significantly reduces misclassifications and improves diagnostic accuracy, offering radiologists a reliable and effective tool to facilitate more accurate evaluations. This may result in prompt identification of conditions, improved therapeutic methods, and improved patient results,the ultimate benchmark of achievement in the field of medical artificial intelligence (AI).

**Table 8:** Classification performance of the proposed model with other state-of-the-art deep learning networks.

Model	Accuracy (%)	Sensitivity (recall) (%)	Specificity (%)	Precision (%)	F1 score (%)
ResNet50	98.39	98.30	98.87	96.50	97.40
InceptionNet	98.55	98.50	99.02	96.83	97.65
MobileNet	98.72	98.60	99.15	97.05	97.80
Inception-ResNet	98.89	98.81	99.28	97.40	98.10
GoogleNet	99.15	99.02	99.40	97.85	98.43
Proposed	99.67	99.60	99.64	99.35	99.60

**Fig. 11:**
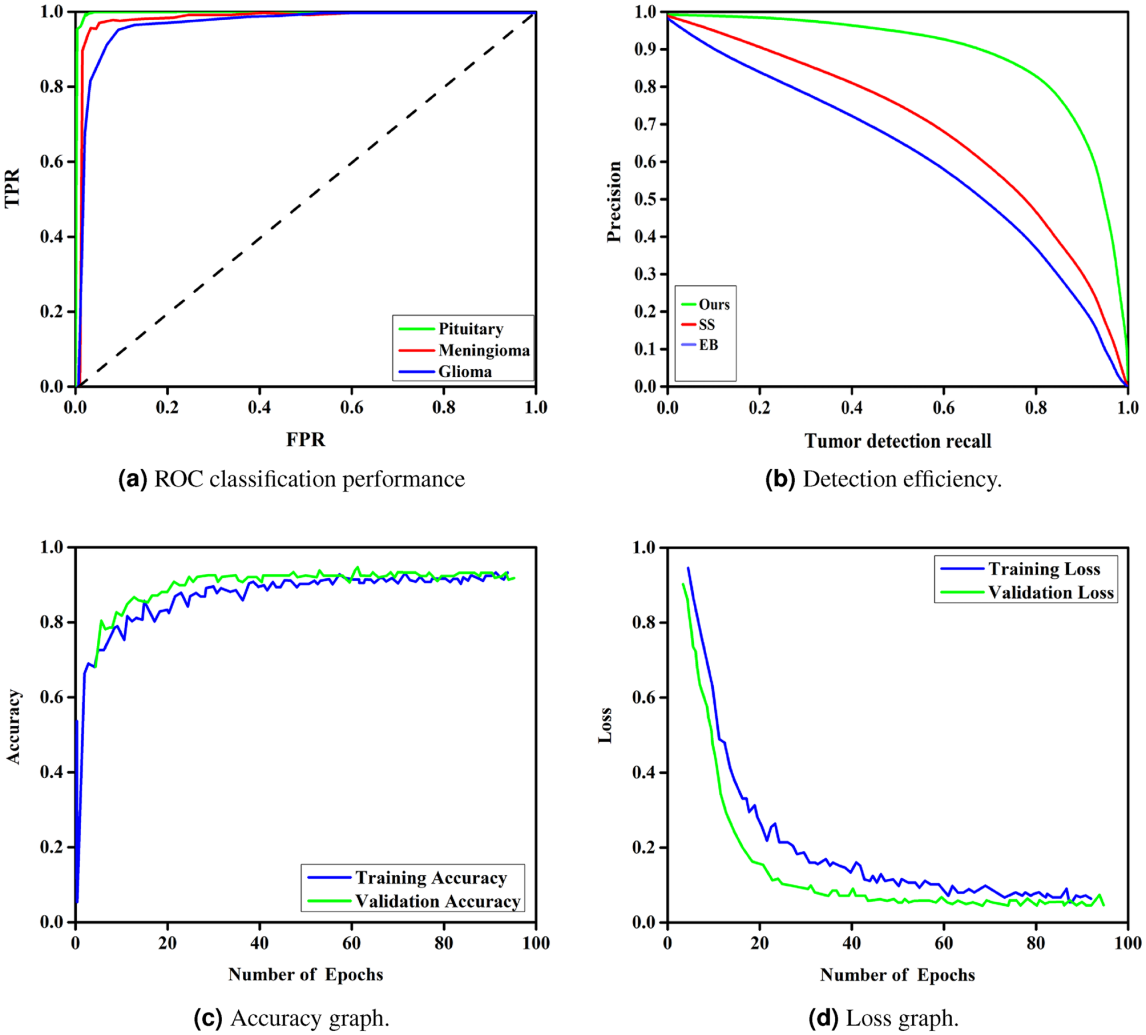
Overall model’s performance.

Table 9 compares the proposed method with a variety of well-established methods to classify three-class brain tumors using the same dataset. The table provides a summary of the classification results based on the standard accuracy metric used in all previous techniques. To guarantee optimal performance, the proposed model was tested based on the factors detailed in Tables 3,  4, and  5. Our method obtained a stunning 99.67% accuracy in only 100 epochs, outperforming other methods without requiring manual segmentation, as seen by the results. This remarkable accuracy demonstrates the effectiveness of our deep learning-based approach for the extraction and classification of brain tumor features. Moreover, our methodology outperformed the competition not only in accuracy, but also in all other quality metrics. Fig. 11a shows the ROC curve of the classification performance of the proposed model, which exhibits excellent results with correlation values of 0.9944, 0.9937, and 0.9978 for the Meningioma, Glioma and Pituitary classes. Compared to other forms of tumor, the Pituitary has the highest rate of true positives. The detection efficiency of our proposed method, based on the average accuracy curve, is shown in Fig. 11b, showing that our bounding box orientation consistently allows tumor detection. Our experiments provided a significant performance improvement, aligning us with the best through hyperparameter optimization and suitable architecture design. However, brain tumor classification remains a difficult task, as it is influenced by several factors. These include tumor shape, orientation, size, low contrast in MRI images, and the small number of training samples, which can increase overfitting and misclassification, thus lowering classification accuracy. Compared to previous approaches, our proposed scheme eliminates these challenges to a large extent while maintaining an acceptable level of accuracy. To further enhance the classification performance, we increased the sample data, improved the contrast, and correctly determined the tumor location before the final classification. This approach elevates our accuracy above that of competitors and differentiates our method from others. As a result, our model achieved great classification results and quickly reached its peak performance, dramatically eliminating the problem of overfitting. In addition, Fig. 11c and d depict the training and validation phases of our network. The accuracy and loss curves illustrate the remarkable performance and consistent training of our model in each stage.

**Table 9:** A comparative evaluation with existing techniques.

Refs.	Methodology	Key features	Segmentation method	No. of images used	Overall accuracy	Evaluation method
^30^	BoW-SVM	BOW	Yes	3064	91.28	Introduced split
^31^	NN	DWT-Gabor	Yes	3064	91.9	Training-validation
^32^	Preprocessing-SVM	2D DWT using Daubechies wavelets base	No	3064	86	10-fold CV
^33^	ConvNet (CNN)	CNN	No	989 (axial only)	84.52	5-fold CV
^33^	ConvNet	CNN	No	989 (axial only)	90.26	5-fold CV
^34^	CapsNet	CNN	Both	3064	86.56 (via segmentation), 72.13 (via raw images)	Not mentioned
^35^	CapsNet	CNN	Bounding box	3064	90.89	Not mentioned
^36^	8Holistic-RNN (LSTM-autoencoder)	Dense CNN	No	989 (axial only)	92.13	Training-validation testing
^37^	ELM	CNN	Not mentioned	3064	93.68	Training-validation
^38^	Different ConvNet	Model-based	No	82100 (700 from each tumor type)	84.19	Training-validation
^39^	GAN-ConvNet	CNN	No	3064	93.01	8Introduced split, 5-fold CV
^40^	PETNet	CNN	No	327	82 classes: 93%, 3 classes: 77%	Not mentioned
^41^	GA-CNN	CNN	No	3064	94.2%	Not mentioned
^42^	CapsNet	CNN	No	3064	94.74	5-fold CV
^43^	DeepCNN-SVM	CNN	No	3064	97.10	5-fold CV
^44^	CNN-SVM	CNN	No	3064	97.10	5-fold CV
^45^	8ResNet-50 Global average pooling	CNN	No	3064	97.48	5-fold CV
^46^	Average pooling	CNN	No	3064	97.42	5-fold CV
^47^	EfficientNet	CNN	Bounding Box	3064	98.04	5-fold CV
^20^	EfficientNet-B0, ResNet50	CNN	Bounding Box	3064	98.95	5-fold CV
^21^	Combined deep features-SVM	CNN	Bounding Box	3064	98.98	5-fold CV
Proposed	Multiscale features-ensemble	CNN	U-Net	3064	99.67	5-fold CV

## Model interpretability, trustworthiness, and generalization across datasets

The efficacy of the proposed method was further evaluated to assess generalizability and robustness between different datasets using the BraTS2020 dataset (https://www.kaggle.com/datasets/awsaf49/brats20-dataset-training-validation)

obtained from the Kaggle database. This dataset comprises 3,929 brain MRI scans, consisting of 2,756 images showing tumors and 1,173 images without tumors. The photographs affected by tumors are classified into two types: 1,290 images illustrate malignant tumors, and the remaining 1,466 portray benign tumors. The images were obtained from 3,929 people between 2005 and 2020 using MRI scanning technology. The dataset was first published in 2015 and subsequently updated in 2021. Augmentation techniques were used to enrich the dataset, resulting in 7858 samples. This dataset is especially appropriate for tumor classification due to its intrinsic imbalance, which helps to evaluate the robustness of the proposed approach.

The evaluation of the proposed model is shown in Table 10.The results of the BraTS2020 dataset highlight the effectiveness of the proposed model in all key metrics compared to existing models such as ResNet50, InceptionNet, MobileNet, Inception-ResNet, and GoogLeNet. The proposed model achieves high performance metrics, including accuracy (99.94%), sensitivity (99.96%), specificity (99.92%), precision (99.96%) and F1-score(99.96%), highlighting its effectiveness in tumor identification and its ability to reduce false positives and negatives. This performance is significant due to the imbalance of the dataset, characterized by more images representing malignancies. ResNet50 demonstrates robust performance; however, sensitivity and F1-score enhancements are necessary. InceptionNet and MobileNet produce similar results, and it is noteworthy that the proposed model outperforms them in all metrics. Although GoogLeNet demonstrates strong performance, the proposed model exceeds all metrics. The findings indicate that the proposed model performs exceptionally well on the Figshare dataset and adapts efficiently to the BraTS2020 dataset, underscoring its considerable potential for practical clinical applications. Future efforts will evaluate the model’s applicability to additional datasets and clinical settings to improve its generalization and practical use.

**Table 10:** Performance comparison of different models for BTC on BraST2020 dataset.

Model	Accuracy	Sensitivity (recall)	Specificity	Precision	F1 score
ResNet50	99.61%	99.41%	98.99%	99.45%	99.43%
InceptionNet	99.62%	99.42%	99.23%	99.59%	99.50%
MobileNet	99.64%	99.59%	99.27%	99.61%	99.60%
Inception-ResNet	99.68%	99.81%	99.46%	99.70%	99.75%
GoogLeNet	99.72%	99.51%	99.64%	99.90%	99.71%
Proposed	99.94%	99.96%	99.92%	99.96%	99.96%

In healthcare technology applications, a primary challenge is ensuring that the system’s decisions are accurate, intelligible, and reliable for medical practitioners. Significant effort has been dedicated to ensuring that the model is transparent rather than an opaque “black box.” The objective is to ensure that physicians have confidence in its reliability and thoroughly understand its performance. Various elements have been incorporated to clarify the rationale underlying the model’s predictions. The integration of attention mechanisms was a critical measure that was implemented. This enables the model to focus on the most important aspects of the MRI images. However, the essential aspect is that they enable physicians to observe precisely on which regions the model focuses when generating its predictions. This clarity is essential because it helps healthcare professionals understand why the model reached specific conclusions. In addition, saliency maps are other methods used. It highlights the areas in the image that played a prominent role in the model’s ability to make decisions. It gives doctors a kind of visual cheat sheet to understand the essential elements of the model. The presented model has been evaluated on different datasets to confirm its robustness and to show that it can produce consistent results across various conditions. Healthcare professionals who had offered their input on the predictions were now involved in a feedback loop. This iteratively enhances the consultative model and lays the path for it to become a trusted and reliable solution. Finally, all of the model’s processes, from training to evaluation, are transparent and comprehensible to medical clinicians. By doing so, the hope is to provide an application that is functional and reliable, so that it can be implemented in real clinical practice.

## Discussion

This study tackles an important problem in medical imaging: correctly classifying brain tumors using MRI scans. Brain tumors can be of many shapes, sizes, and locations where they occur. Their timely diagnosis is the key to effective treatment planning and better clinical results. This task has traditionally been performed by radiologists through image analysis, a process that is slow, open to human error, and varies from practitioner to practitioner. To address these difficulties, we developed an automated categorization framework that aimed to increase the accuracy and reliability of the diagnosis while being much less dependent on humanization.

The experimental results validate the effectiveness of the proposed methodology. As shown in Table 2, diversity is evident in the dataset. It includes MRI images of the Glioma, Meningioma, and Pituitary tumor from the coronal, sagittal, and transverse planes. This and other types of creative diversity are important in helping the model train on a wide range of cases, boosting its generalizability.

Table 3 presents a comparative analysis of the optimization algorithms, with Adagrad achieving the highest accuracy (99.67%) at a learning rate of 0.003, while SGD and Adam demonstrate strong performance under specific conditions. These findings underscore the importance of hyperparameter tuning in optimizing model performance. Similarly, Table 4 reveals that the model reaches its maximum accuracy (99.67%) at 100 epochs, after which the performance slightly decreases, probably due to overfitting. Table 5 further demonstrates the impact of regularization, with a dropout rate of 0.5 that yields the best accuracy (99.67%), highlighting the need to balance the complexity of the modle and the prevention of overfitting.

The classification performance, as shown in Table 6, is highly robust, with minimal misclassification between tumor types. In particular, of the 704 cases of Meningioma, only four instances were misclassified, and of the 1,426 cases of Glioma, only nine misclassifications were observed. Table 7 further confirms the efficacy of the model, achieving an overall accuracy of 99.67%, sensitivity of 99.60%, specificity of 99.64%, precision of 99.35%, and an F1-score of 99.60%. These metrics indicate that the model is highly reliable, well-balanced, and effective in identifying true positives and minimizing false negatives.

In comparative performance analysis, as shown in Table 8, the proposed methodology consistently outperforms state-of-the-art deep learning models, such as ResNet50, InceptionNet, and GoogLeNet, achieving an accuracy of 99.67%. Further validation on the BraTS2020 dataset Table 10 demonstrates even stronger performance, with an accuracy of 99.94%, reinforcing the robustness and adaptability of the model across different datasets. Table 9 further supports these results, illustrating that our approach surpasses previous methodologies, including BoW-SVM, CapsNet, and DeepCNN-SVM, particularly in terms of automation and classification accuracy.

Despite these promising results, certain limitations must be acknowledged. One key challenge lies in the preprocessing steps required for filtering and morphological analysis, which add complexity to the overall workflow. Further work will focus on refining and optimizing the model to create an end-to-end framework that performs preprocessing, segmentation, and classification in one step. A second constraint is the computational cost of adopting an ensemble learning methodology that requires high-performance hardware and, thus, may be challenging to employ in a resource-limited context. Moreover, despite the fact that the datasets we used in this study are extensive and diverse, they do not represent all the diversity of tumors presented in clinical practice. The model could be improved with generalizability and clinical applicability by extending the datasets to include more rare tumor types, demographic data from patients, and larger sample sizes.

Many aspects of current research directions can be improved, and there are exciting opportunities to do so. Multimodal medical imaging, such as the fusion of CT with standard PET images, though more expensive, would offer additional diagnostic information, which allows the system to be even more generalizable and more cross-diagnostic. Instead of 2D slices, 3D imaging techniques have the potential to provide a better tumor morphology and size. It could offer even greater diagnostic precision. Another primary direction for future work is to increase the interpretability of the model. Deep learning models are often “black boxes”, keeping clinicians in the dark about how decisions are made for specific patients. We would integrate the attention map and the saliency map to justify the predictions made by the model, which would help build more trust and adoption among medical professionals. The techniques developed in this work can be adapted to other areas of medical diagnostics. AI-enabled techniques like this can even be custom-built to spot potential malignancies for breast cancer, lung nodules, liver lesions, and numerous other forms of cancer, illustrating an area of health services that may potentially be immense. By continually refining and adapting these technologies, AI-driven medical diagnostics can play a pivotal role in helping physicians, improving patient outcomes, and transforming modern healthcare.

Ultimately, while challenges remain, such as computational constraints, dataset variability, and the need for greater interpretability, this study represents a significant step forward in AI-powered BTC. By bridging the gap between technology and clinical expertise, this research paves the way for a future where AI improves the accuracy, efficiency, and accessibility of medical diagnostics, ultimately improving patient care and saving lives.

## Conclusion

Extensive research has been conducted on BTC in recent years. By introducing various classification methods, an adequate level of accuracy has been achieved in classifying different malignancies. The classification of cancers remains ambiguous, and there is always room for new research. Even greater classification accuracy can be achieved by employing a pragmatic framework. This study introduces a method to classify brain lesions using MRI images. Initially, the visual quality of images is improved by filtering techniques, which combine AGSW with guided filtering. These methods facilitate the identification of tumor regions by sharpening images and reducing noise. Morphological analysis was used to eliminate irrelevant areas, thereby directing the attention of the model solely to the significant regions of the image. The images were segmented using a deep neural network, which led to the acquisition of high-quality ROIs. A multiscale technique is then used to extract features, simplify complex data processing, and efficiently manage anomalies. To ensure that the model remained focused on essential aspects, an attention mechanism that functions as a spotlight is incorporated to emphasize the most salient characteristics of the tumor. Finally, to determine the category of the tumor in images, RF, SVM and KNN classification models integrated into an ensemble. This enables the network to capitalize on the advantages of each model and produce more reliable predictions. Implementing data augmentation strategies reduces the incidence of network overfitting. Two datasets that are readily accessible on Figshare and Kaggle were used to validate the proposed model. Compared with other procedures of comparable nature, the experiments yielded consistent results. The proposed methodology obtains a precise classification rate of 99.67% for Figshare and 99.94% for Kaggle (BraST2020) datasets, surpassing previous research using the same dataset. However, precision alone cannot fully convey the complete story. The model also demonstrated excellent performance in several key metrics, leading to balanced and highly reliable classification performance. This proposed approach eliminates the need for manual segmentation of the lesion prior to classification, resulting in increased speed and resilience. The proposed procedure appears to be a suitable classification tool for BTC. The proposed system is expected to contribute considerably to the preservation of lives and provide a high level of precision in the diagnosis of brain tumors once it is operational.

## Data Availability

The datasets used and/or analysed during the current study are available from the corresponding author on reasonable request.
